# GMMA as adjuvant and carrier for mucosal delivery of group A streptococcus glycoconjugate vaccine

**DOI:** 10.1080/21645515.2026.2740963

**Published:** 2026-10-19

**Authors:** James Ozanne, Manolya Saydam, Elena Palmieri, Roberta Di Benedetto, Anjum Aktar, Martina Carducci, Luca Rovetini, Omar Rossi, H. Alexandra Shaw, Francesca Micoli, Fatme Mawas

**Affiliations:** a Vaccines Division, Science & Research, Medicines and Healthcare Products Regulatory Agency, Potters Bar, UK; b GSK Vaccines Institute for Global Health Srl, Siena, Italy

**Keywords:** Group A streptococcus, Generalized Modules for Membrane Antigens (GMMA), conjugate vaccine, mucosal immunity

## Abstract

Group A streptococcus (GAS) infection is usually initiated by colonization of mucosal sites such as the pharynx, followed by invasion and systemic dissemination. Development of a vaccine that can prevent colonization is enticing as it would prevent subsequent infections and all associated autoimmune sequalae such as rheumatic heart disease (RHD). In this study we investigated the mucosal adjuvanticity of GMMA when used as a carrier protein for GAS carbohydrate (GAC) conjugate vaccine (GAC-GMMA). Mice were immunized sublingually or intranasally with GAC-GMMA or GAC-CRM_197_, in the presence or absence of cholera toxin B subunit (CTB). The anti-GAC systemic and mucosal immune responses were evaluated and compared. Our results showed that, in addition to being a good carrier, GMMA is also a good mucosal adjuvant and mediated the induction of substantial levels of serum IgG median titer: 1,050–3,200) and of mucosal IgA (512–2,560) in the mouth, (640) in the nose, (768–1,024) in the intestines and (1,024–1,280) in the vagina. The induced response was similar or better than the response induced by the GAC-CRM_197_ conjugate administered in combination with the mucosal adjuvant CTB. Furthermore, mucosal delivery of GAC-GMMA and GAC-CRM_197_ induced IL-17 and IFNγ secretion by immune spleen cells, which are known to mediate neutrophils recruitment and nitric oxide production by macrophages necessary for controlling infections at mucosal surfaces. We demonstrated that GMMA could work both as a carrier and adjuvant for the mucosal delivery of GAC and mediates induction of both systemic and mucosal immune responses against GAS.

## Introduction

Group A streptococcus (GAS), or *Streptococcus pyogenes*, is a major global pathogen. Initial infection through mucosal sites can manifest as pharyngitis in the throat, or as impetigo through infection of the skin, and lead to more serious systemic infections such as toxic shock syndrome and necrotizing fasciitis.^1^ The most significant impact of GAS infection globally derives from secondary diseases caused by autoimmune sequelae, such as rheumatic heart disease (RHD), which accounts for significant Disease Adjusted Life Years (DALYs), especially in low- to middle-income countries (LMICs).^2,3^ The incidence of RHD has been attributed to repeated mild mucosal and skin infections, therefore, it has been highlighted by the WHO as an imperative of vaccines to prevent mucosal infections such as pharyngitis as an initial step toward preventing RHD.^4^ Due to the impact of RHD in LMICs, it would be ideal for a vaccine targeting GAS to be cost-effective to produce and deliver.

Disease progression is caused by multiple pathogen virulence factors, which has hindered vaccine development.^5^ The most advanced vaccine candidates target the M-protein, a surface exposed protein which is the major virulence factor of GAS infections.^6,7^ The M-protein is limited by its sequence variability, and complications arise when using the conserved regions near the cell wall binding domain of the protein. Other multicomponent vaccines have targeted more conserved proteins that play roles in different stages of infection, as these proteins have different expression profiles in different diseases and across serotypes.^8–12^ The Group A Carbohydrate (GAC) is a conserved polysaccharide that makes up more than half of the cell wall mass. Its ubiquitous nature and conservation make it an ideal vaccine candidate to target all serotypes and disease phenotypes.^13^ The prevalence of circulating antibodies against GAC in Mexican children was shown to inversely correlate with pharyngeal colonization, highlighting anti-GAC antibodies as a potential prevention of mucosal infection.^14^ Candidate GAC (or derivative) conjugate vaccines have been prepared using classic carrier proteins like tetanus toxoid (TT)^14^ or CRM_197_, a non-toxic mutant of diphtheria toxin^15,16^ as well as native GAS proteins such as SpyAD,^17^ C5a peptidase^18^ and IdeS.^19^ These have been shown to induce antibodies in animal models in a T-dependent (TD) manner. Parenteral immunization with a TT-GAC conjugate induced protection in a mouse challenge model of systemic infection using two different M serotype GAS strains.^16^ GAC and rhamnose derivatives have been shown as effective conjugate components in multiple studies with novel technologies such as gold nanoparticles and self-adjuvanting liposomes,^13,20^ More recently, GAC conjugate has been used in a multicomponent GAS vaccine candidate (from GSK Vaccines Institute for Global Health; GVGH), which contains GAC-CRM_197_ conjugate and other conserved antigens (SLO, SpyCEP and SpyAD) and is currently entering Phase I clinical trial, after promising data from preclinical studies.^21^ There has been controversy over the GlcNAC side chains of GAC potentially being implicated in autoimmune responses seen following repeated exposure to GAS, but a recent publication of a thorough analysis of the literature concluded that GAC was unlikely to be responsible for this.^22^

Generalized Modules for Membrane Antigens (GMMA) are a novel approach to vaccine delivery that exploit the naturally occurring membrane blebs from Gram‑negative bacteria.^23^ GMMA producer strains are genetically modified to induce hyper-blebbing and reduce the endotoxic activity of the native lipopolysaccharides. GMMA manufacturing is simple and particularly attractive for the development of cost-effective vaccines for LMICs.^24^ O-antigen-based vaccines against *Shigella* and *Salmonella* are currently being tested in Phase 2 clinical trials.^25,26^ GMMA have also been demonstrated as effective carriers for heterologous polysaccharides.^27^ We have recently demonstrated that GMMA, due to their intrinsic adjuvant properties, could work both as carrier and adjuvant for GAC.^28^ Further, subcutaneous (SC) immunization with a GAC-GMMA conjugate induced a similar or higher level of antibody response to GAC compared to the traditional CRM_197_ carrier protein, even when the CRM_197_ conjugate was used with adjuvant.^27^ However, the capacity of GMMA to induce mucosal immune responses, particularly in the context of glycoconjugate vaccines, remains incompletely understood.

GAS infection usually starts by colonization of mucosal sites such as the pharynx, followed by invasion and systemic dissemination. To date, most successful bacterial vaccines are administered intramuscularly and induce strong serum IgG responses. These vaccines, including those against *Streptococcus pneumoniae*, *Haemophilus influenzae* type b, and *Neisseria meningitidis*, have demonstrated the ability to significantly reduce mucosal colonization and transmission, highlighting the effectiveness of systemic immunization in protecting mucosal sites.^29–31^

Although the role of mucosal vaccines in protecting against diseases is still an area of ongoing investigation, it may offer advantages over parenteral immunization, including the induction of both systemic and local immune responses at the site of pathogen entry, such as secretory IgA, as well as potential logistical benefits related to ease of administration.^32^ However, clear evidence demonstrating the superiority of mucosal over systemic vaccination for bacterial pathogens in humans is currently lacking. In this context, preclinical studies provide an opportunity to explore the mechanisms of mucosal immune induction and to evaluate novel vaccine platforms.

Development of a vaccine that can prevent GAS colonization is particularly attractive as it could avert subsequent infections and associated autoimmune sequalae such as RHD.^32,33^ This would also meet the WHO preferred product characteristics (prevention of pharyngitis).^4^ Once a GAS vaccine is licensed, it is likely to be incorporated into childhood immunization programs. The success of intranasal live-attenuated influenza vaccines, which demonstrate comparable efficacy to inactivated intramuscular vaccines and induce mucosal immunity,^34^ motivated us to investigate the immunogenicity of the GAC-GMMA vaccine following mucosal delivery.

The aim of this work is to expand on our previous work to assess the immunogenicity of a GAC-GMMA conjugate vaccine following mucosal delivery via the sublingual (SL) and intranasal (IN) routes in a mouse model and to contribute to the broader understanding of how such platforms may be leveraged in vaccine design. Particularly of ongoing interest is the ability for GMMA to be both vaccine carrier and adjuvant, reducing the need for additional components in a vaccine. The immune responses elicited by GAC-GMMA were compared with those induced by a classical conjugate vaccine, GAC-CRM_197_ conjugate, administered in the presence or absence of cholera toxin B subunit (CTB), a widely used experimental mucosal adjuvant.

## Materials and methods

### Materials

GAS carbohydrate (GAC) conjugated to either CRM_197_, a non-toxic mutant of diphtheria toxin, or GMMA produced from *Salmonella* Typhimurium lacking the O antigen component of LPS, were synthesized and characterized as described previously.^28^ All chemicals and reagents used in this study were of analytical grade and purchased from Sigma‑Aldrich unless stated otherwise.

**Table:** 

Vaccine	Conjugation chemistry	PS: Protein (w:w ratio)	% free PS
GAC-CRM_197_	Reductive amination	0.5	<12
GAC-GMMA	Reductive amination	5	<6.5

### Animals and immunization

Six- to twelve-week-old female BALB/c mice (Charles River, UK) were used. Animals were non-genetically modified, had not been used in previous procedures and were acclimatized to the animal facility for at least 7 d prior to immunization. Animals were housed under standard conditions in the MHRA Animal Care Facility with ad libitum access to food and water and were monitored daily by trained animal care staff. Mice were randomly allocated to treatment groups by a laboratory technician not involved in downstream analyses. Exclusion criteria were defined a priori and included unanticipated death, or signs of pain or distress meeting humane endpoint criteria.

Animals were anesthetized in an isoflurane chamber, using 5% for induction and 1.5–3% for maintenance in oxygen. Once anesthetized, mice were immunized (once a week for five weeks) either sublingually (20 µl) or intranasally (10 µl; 5 in each nostril) with GAC conjugated to either GMMA or CRM_197_ (containing 2 µg of GAC; pre-determined to be the optimal dose in a preliminary experiment), with or without 2 µg of the Cholera toxin B subunit (CTB; Sigma-Aldrich). Animals immunized with phosphate-buffered saline (PBS) served as the negative control group. The liquid vaccine was deposited slowly and carefully onto the underside of the tongue or to the entrance of the nasal cavities using a micropipette in a controlled manner to promote slow interaction with the mucosa. Once the entire dose is dispensed (and for intranasal, taken into the nostrils), the mouse is returned to the anesthezia box and placed in a dorsal uppermost position with head straightened to prevent airway obstruction. The mouse remains under anesthezia for a further 30 min before being allowed to recover. Subcutaneous (SC) immunization (once a week for five weeks) with 200 µl of the GAC-GMMA conjugate or with GAC-CRM_197_ conjugate adsorbed on Alhydrogel (alum) (containing 2 µg of GAC) served as the positive control for induction of serum IgG response. At the end of the experiment, the mice were terminally bled by cardiac puncture under general anesthesia as follows: mice were administered pentobarbital (10%, 130–150 μL per animal) via intraperitoneal injection to induce deep anesthesia. Mucosal secretions (intestinal, vaginal, nasal and mouth washes) were collected in protease inhibitor buffer (Fisher Scientific UK), supplemented with 2% Fetal calf serum (FCS). For intestinal washes, the small intestines were collected in 3 mL of protease inhibitor buffer, chopped into small pieces, mixed thoroughly, centrifuged and supernatant collected. Oral, nasal, and vaginal washes were obtained by rinsing mucosal surfaces twice with 50 µL protease inhibitor buffer. All samples were stored frozen until assessed.

All animal procedures were conducted in accordance with the Home Office (Scientific Procedures) Act 1986.

### ELISA for IgG and IgA anti-GAC antibodies

An in-house ELISA was used to determine the level of GAC-specific IgG or IgA antibodies, as previously described,^27^ with GAC- specific IgG or IgA detected using rabbit anti-mouse IgG or IgA antibodies conjugated to horse radish peroxidase (HRP), (Sigma‑Aldrich; A4416 and A4789, respectively). Data are presented as antibody titer of each animal and as the median titer with interquartile range (IQR) for each group. The antibody titer was determined as the last dilution giving OD of >0.5 for IgG and as the endpoint titer (the last dilution giving an OD > mean Blank OD + 3SD) for IgA. Samples with titers below the limit of detection were assigned a value of 50 for IgG and 1 for IgA for data analysis and graphical presentation.

### Immunofluorescence staining and flow cytometry

To measure anti-GAC serum IgG and intestinal IgA antibodies binding to GAS the M1 strain NCTC 8198 was used, using immunofluorescence staining as described before,^27^ with goat anti-mouse IgG-FITC (ThermoFisher Scientific, Waltham, MA, USA) or goat anti-mouse IgA-FITC (Abcam, Cambridge, UK) as the detection antibodies. Results are presented as fluorescence histograms with the fluorescence intensity displayed on the X axis and the percentage of the maximum number of events on the Y axis.

### Cytokine production in immune spleen cells

Spleens were removed at the end of the experiment and single cell suspensions were prepared and stored frozen in liquid nitrogen until used. On the day of the assay, cells were resuscitated and washed x3 with Hank’s balanced salt solution (HBSS), supplemented with 5% FCS, 100 U of penicillin/ml, and 100 µg/ml of streptomycin. Cells were cultured in 24-well plate for 2 d in the presence of 5 µg/ml of GAC-CRM_197_ or GAC-GMMA in a total volume of 2 ml of RPMI 1640, supplemented with 10% fetal calf serum, 2 mM L-glutamine, 50 mM 2-mercaptoethanol, 100 U of penicillin/ml, and 100 µg/ml of streptomycin. At 48 hours, the levels of IL-4, IFNγ and IL-17 in the supernatants of stimulated cells were measured by standard sandwich ELISAs (Invitrogen). The cytokine concentration was calculated relative to each cytokine reference standard, tested on the same plate. The assay cut-off was 4 pg/ml for IL-4 and IL-17 and 16 pg/ml for IFNγ.

### Statistical analysis

Statistical analyses for antibody titers were performed using GraphPad Prism (version 11) Individual mouse antibody titers are shown, and data are presented as median and interquartile range (IQR). Overall differences among groups were assessed using the Kruskal-Wallis test. Pairwise comparisons were performed using two-tailed Mann-Whitney U tests. Effect sizes were calculated as rank-biserial correlations (*r*rb), with values of approximately 0.1, 0.3, and 0.5 were considered small, moderate, and large effects, respectively. Differences were considered statistically significant at *p* < .05.

## Results

### Immunogenicity of GAC-GMMA by the sublingual route

In a first experiment, mice immunized sublingually with GAC-GMMA conjugate (once a week for 5 weeks) mounted a robust systemic anti-GAC IgG response in all immunized animals (Figure 1(a)), with a median antibody titer of 1,050 (425–2,900), compared with 100 (50–110) in the PBS-immunized group (*p* = .0016). The very large effect size (*r*rb = 1.00) indicated complete separation of responses between the groups.

**Figure 1.**
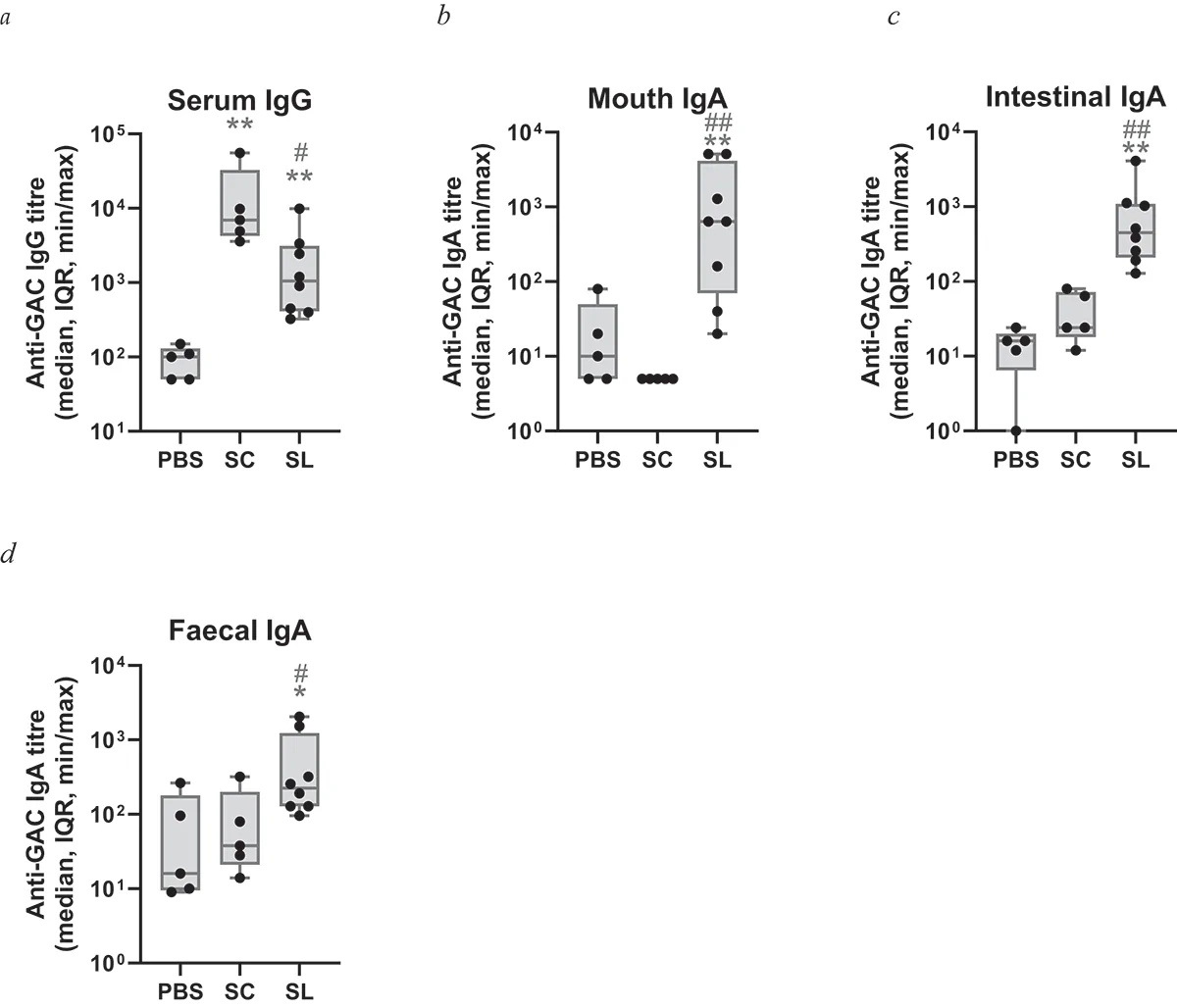
Immunogenicity of GAC-GMMA following sublingual immunization. Groups of mice were immunized sublingually five times, at weekly intervals, with GAC-GMMA conjugate (n = 8) or with PBS (n = 5). Control mice (n = 5) were immunized subcutaneously with GAC-GMMA. Individual animal responses are shown as dots. Horizontal lines indicate the median and boxes represent the interquartile range (IQR). Serum IgG (a), mucosal IgA in mouth (b), intestines (c) and fecal pellets (d). Overall differences between groups were assessed using the Kruskal-Wallis test (*p* < .0001 for serum IgG, mouth IgA and intestinal IgA; *p* = .025 for fecal IgA). Significant differences compared with the PBS group are indicated by *p < .05 and **p < .01; significant differences) between active immunization groups are indicated by #*p* < .05 and ##*p* < .001).

Subcutaneous (SC) immunization induced the highest antibody response, with a median titer of 6,950 (4,900–9,800), and the titers were significantly greater than those observed in the PBS group (*p* = .0079; rrb = 1.00). Titers were significantly higher following subcutaneous than sublingual immunization (*p* = .02; rrb = 0.8), indicating a large difference in response magnitude between the groups.

SL but not SC immunization with GAC-GMMA elicited robust mucosal anti-GAC IgA responses at, both the local (mouth) and distal (intestines and fecal) mucosal sites (Figure 1(b-d)). In the oral cavity, IgA titers reached a median of 640 (100–3200), compared with 10 (5–20) in PBS-immunized mice (*p* = .0047) with a very large effect size (rrb = 1.00). Similarly, intestinal IgA titers reached a median titer of 448 (176–892), which was significantly higher than the PBS control group ((*p* = .002, rrb = 1.00). Fecal IgA titers were also high following SL immunization, reaching a median of 192 (128–896), compared with 16 (12–40) in the PBS group (*p* = .014; rrb = 0.90). Collectively, these findings demonstrate that SL administration of GAC-GMMA induced robust mucosal IgA responses across multiple mucosal compartments.

As fecal IgA served as a noninvasive surrogate marker of mucosal antibody responses, titers were also measured after three immunizations to determine whether a shortened regimen could induce comparable responses. Following three doses, fecal IgA titers remained low with a median of 128 (80–256) and were comparable to those observed in PBS-treated controls 257 (145–391). These findings indicate that three immunizations were insufficient to induce the enhanced mucosal IgA responses observed following the five-dose SL immunization schedule (data not shown).

*Mucosal adjuvant does not improve systemic or mucosal immune responses to sublingually delivered GAC-GMMA, but is required for GAC-CRM_197_ conjugate*.

In a second experiment, we investigated whether the systemic and mucosal antibody responses induced by SL immunization with GAC-GMMA could be further enhanced by the inclusion of the mucosal adjuvant cholera toxin B subunit (CTB). The effect of CTB on the response induced by the classical GAC-CRM_197_ conjugate was also investigated. Vaginal IgA response was included as an additional distal mucosal site. SC immunization with GAC-GMMA or GAC-CRM_197_ adsorbed to alum served as positive controls for the induction of systemic IgG responses and negative controls for the induction of mucosal IgA responses. SL immunization with GAC-GMMA induced robust anti-GAC IgG response (Figure 2) with a median titer of 3,200 (800–4,800), compared with 37.5 (37.5–50) in PBS -immunized mice (*p* = .008, rrb = 0.88). The addition of CTB did not enhance the response to GAC-GMMA. Indeed, mice immunized with GAC-GMMA+CTB exhibited median IgG titer of 800 (400–800 l) which was lower than those receiving GAC-GMMA, although this difference was not statistically significant (*p* = .294; rrb = 0.44).

**Figure 2.**
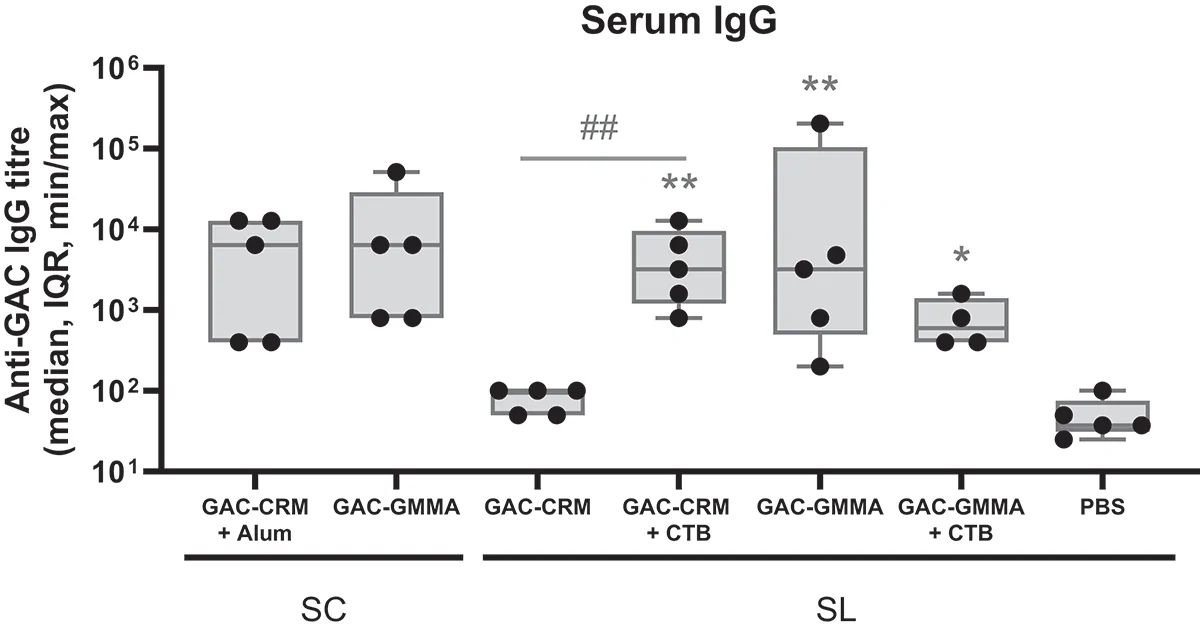
Effect of CTB adjuvant on the IgG response to GAC-GMMA and GAC-CRM_197_ conjugates following SL immunization. Groups of mice were immunized sublingually five times, at weekly intervals, with GAC-GMMA conjugate (with or without CTB), GAC-CRM_197_ (with or without CTB), or with PBS. Control mice were immunized subcutaneously with GAC-GMMA or GAC-CRM197/alum. Individual animal responses are shown as dots. Horizontal lines indicate the median and boxes represent the interquartile range (IQR. Overall differences between groups were assessed using the Kruskal-Wallis test (*p* = .0059). Significant differences with the PBS control group are indicated by *p < .05; **p < .01; significant differences between groups with and without CTB are indicated by ##*p* < .01.

In contrast, CTB substantially improved the immunogenicity of GAC-CRM_197_ following SL administration. Mice immunized with GAC-CRM_197_ alone generated low anti-GAC IgG responses that were comparable to those observed in PBS-immunized controls 100 (50–100); *p* = .135). However, inclusion of CTB induced a significant response with a median titer of 3,200 (1,600–6,400); (*p* = .008, rrb = 1.00). These responses were comparable to those induced by SL administration of GAC-GMMA (*p* = .810; rrb = 0.12). Although higher median titers were observed in the SC positive control groups (6400 for both GAC-GMMA and alum-adjuvanted GAC-CRM_197_), these comparisons were not included in the statistical analysis and are therefore presented descriptively only.

Similar trends were observed when mucosal IgA responses were measured at both local (Figure 3(a)) and distal mucosal sites (intestinal and vaginal; (Figure 3(b,c)), where the addition of CTB did not enhance mucosal IgA responses in any compartment examined. In the oral cavity, the median oral IgA titer in mice immunized with GAC-GMMA alone 512 (256–2,048) was higher than in those receiving GAC-GMMA+CTB 192 (128–256), but the difference was not statistically significant (*p* = .262) and was associated with a moderate effect size (rrb = 0.45), providing no evidence that CTB enhances oral IgA responses induced by GAC-GMMA.

**Figure 3.**
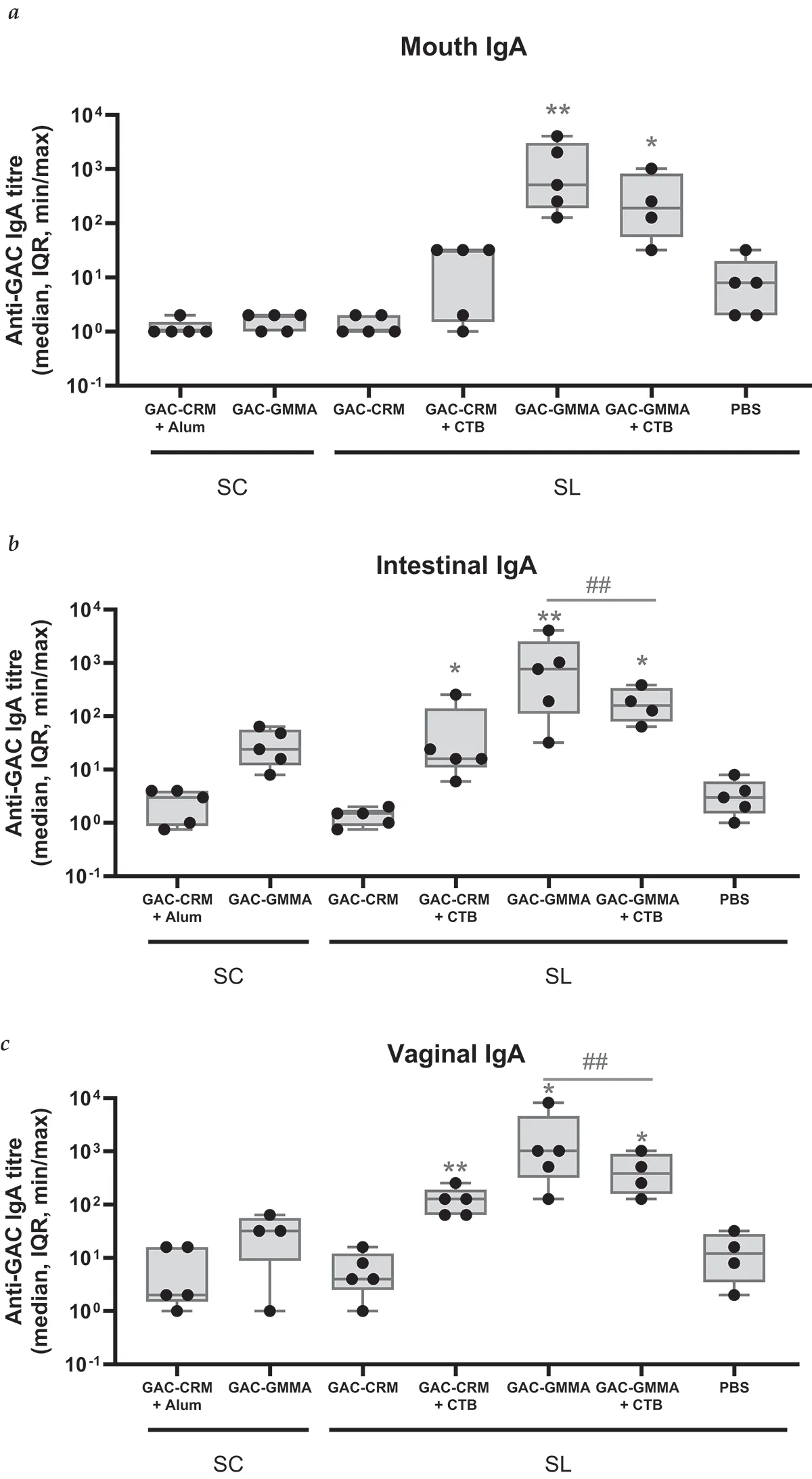
Effect of CTB adjuvant on the IgA response to GAC-GMMA and GAC-CRM_197_ conjugates following SL immunization. Groups of mice were immunized sublingually five times, at weekly intervals, with GAC-GMMA conjugate (with or without CTB), GAC-CRM_197_ (with or without CTB), or with PBS. Control mice were immunized subcutaneously with GAC-GMMA or GAC-CRM_197_ /alum. Individual animal responses are shown as dots. Horizontal lines indicate the median and boxes represent the interquartile range (IQR). Data is presented for anti-GAC mucosal IgA (a) mouth, (b), intestines, and (c) vagina. Overall differences between groups were assessed using the Kruskal-Wallis test (*p* = .0024 for mouth IgA; *p* = .003 for intestinal IgA and *p* = .0027 for fecal IgA. Significant differences compared with the PBS group are indicated by *p < .05 and **p < .01; significant differences between groups with and without CTB are indicated by #*p* < .05 and ##*p* < .01.

Similarly, intestinal anti-GAC IgA titers were higher in the GAC-GMMA group 768 (192–1,024) than in the GAC-GMMA+CTB group 160 (112–240) but the difference was not statistically significant (*p* = .318; rrb = 0.45). Vaginal IgA responses followed the same pattern, with median titers of 1,024 (512–1,024) and 384 (256–512) observed in the GAC-GMMA and GAC-GMMA+CTB groups, respectively, (*p* = .389; rrb = 0.50). Collectively, these findings provide no evidence that CTB enhances mucosal IgA responses induced by GAC-GMMA following SL immunization, suggesting that the intrinsic immunostimulatory properties of the GMMA are sufficient to promote mucosal IgA responses without additional adjuvant.

In contrast, higher mucosal IgA titers were observed in mice immunized with GAC-CRM_197_ in the presence of CTB than in those immunized with conjugate alone following SL immunization (Figure 3). In the oral cavity, mice immunized with GAC-CRM_197_ alone developed no IgA responses, with a median titer of 1 (1–2), whereas the addition of CTB increased titers to 32 (2–32); however, the difference did not reach statistical significance (*p* = .143). Nevertheless, the comparison yielded a large effect size (rank-biserial correlation, rrb = 0.64), indicating a potentially biologically meaningful enhancement associated with CTB co-administration. A similar pattern was observed in the intestinal compartment, where median IgA titers increased from 1.5 (1–1.5) to 16 (16–24) following formulation with CTB (*p* = .008; rrb = 1.00). Vaginal IgA responses were also markedly enhanced, increasing from 4 (4–8) in mice immunized with GAC-CRM_197_ alone to 128 (64–128) in mice receiving GAC-CRM_197_+CTB (*p* = .008; rrb = 1.00). Across all mucosal compartments examined, the inclusion of CTB was associated with higher mucosal IgA titers in mice immunized with GAC-CRM_197_ than in those receiving GAC-CRM_197_ alone. Collectively, these findings suggest that the adjuvant activity of CTB may depend on the vaccine platform with which it is administered.

### GMMA conjugate induces a mixture of Th1 and Th2 response with a bias to Th1

Investigation into the subclass of IgG response induced following SL immunization showed that while GAC-GMMA induced a mixture of IgG1 and IgG2a (Table 1) with a bias to a higher IgG2a response (IgG2a/IgG1 ratio of 3.7), the CTB-adjuvanted GAC-CRM_197_, induced mainly IgG1 response with very little IgG2a (IgG2a/IgG1 ratio 0.01). Interestingly, the GAC-GMMA + CTB group induced a more balanced Th1 and th2 response with an IgG2a/IgG1 ratio of 1.14, demonstrating the synergistic effects of GMMA and CTB.

**Table 1.** IgG1 and IgG2a in mice sera generated by GAC-GMMA and adjuvanted GAC-CRM_197_ following SL immunization.

Relative concentration in ng/ml ± SEM			Average ratio ± SEM
Group	IgG1	IgG2a	IgG2a:IgG1 Ratio
GAC-CRM_197_ +CTB	3,544 ± 1,282	41 ± 29	0.011 ± 0.005
GAC-GMMA	1,219 ± 483	7,184 ± 6,890	3.7 ± 2.53
Significance between groups	*p* > .05	*p* < .05	*p* < .05

Results are presented as mean relative concentration of the group (ng/ml) ± SEM and mean ratio of IgG2a: IgG1. The Mann-Whitney two‑tailed test was performed to compare two groups.

### GMMA is also an effective adjuvant by the intranasal route

Having shown that GMMA was an effective mucosal vaccine delivery platform using the SL route, we wished to explore whether it is also effective when administered by the IN route (already in use for some licensed vaccines). Mice were immunized with GAC-GMMA or GAC-CRM_197_ +CTB via the IN route and systemic IgG as well as mucosal IgA at local (nasal) and distal (mouth, intestinal, and vaginal) mucosal sites were evaluated and compared to the response induced by the SL route. As shown in Figure 4, similar to the SL route, IN immunization with GAC-GMMA induced a significant level of serum anti-GAC IgG antibodies, with median titer 1100 600–2650); (*p* = .008; rrb = 1.00), which was lower than the median response induced by the SL route, 4,000 (3,000–5,000). Although a large effect size was observed for this comparison (rrb = 0.68), the difference between the IN and SL groups did not reach statistical significance (*p* = .079).

**Figure 4.**
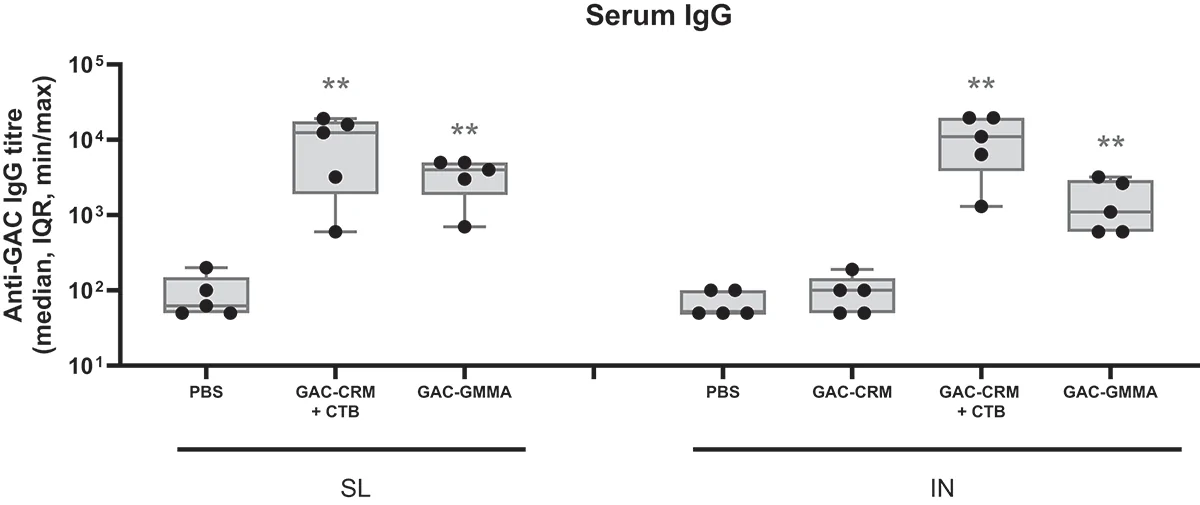
Comparison of serum IgG response following SL or IN immunization. Groups of mice (n = 5) were immunized sublingually or intranasally five times, at weekly intervals, with GAC-GMMA conjugate, GAC-CRM_197_ (with or without CTB), or with PBS. Individual animal responses are shown as dots. Horizontal lines indicate the median and boxes represent the interquartile range (IQR). Overall differences between groups were assessed using the Kruskal-Wallis test (*p* < .0001). Significant differences with the PBS control group are indicated by *p < .05; **p < .01.

Similarly, GAC-CRM_197_+CTB elicited strong systemic IgG responses following both routes of administration, with SL and IN immunization inducing median titers of 12,400 (3,200–15,900) and 11,000 (6,400–19,500), respectively. No significant difference was observed between routes (*p* = .635; rrb = 0.20), indicating comparable systemic immunogenicity following either mucosal delivery approach.

IN immunization with GAC-GMMA induced a significant anti-GAC IgA response in the nasal cavity, the primary site of antigen delivery (Figure 5(a)). Nasal IgA titers reached a median titer of 640 (80–1,280), compared with 5 (5–5) in PBS-treated controls (*p* = .008; rrb = 0.96). Although nasal IgA titers were numerically lower than those observed following SL immunization with GAC-GMMA 1,280 (640–1,280), comparison of the two immunization routes revealed no significant difference in nasal IgA responses (*p* = .983), with only a small effect size observed between routes (rrb = 0.12), demonstrating that GAC-GMMA effectively promotes nasal mucosal immunity following IN and SL administration. Similarly, a substantial oral mucosal IgA response was also observed following IN immunization (Figure 5(b)) with median IgA titer reaching 2560 (1280–2560); (*p* = .008, rrb = 1.00). Oral titers were numerically higher than those observed following SL administration [640 (640–1280)], although the difference between routes was not statistically significant (*p* = .365; rrb = 0.40). Intestinal IgA responses were comparable between routes (Figure 5(c)), with median titers of 1024 (1024–1024) and 1024 (1024–2048) following IN and SL immunization, respectively, and a negligible route effect (rrb = 0.08). Vaginal IgA responses were also readily detected following both routes of administration (Figure 5(d)), with median titers of 1280 (80–2560) following IN immunization and 2560 (1280–5120) following SL immunization. No statistically significant difference was observed between routes (*p* = .246; rrb = 0.24).

**Figure 5.**
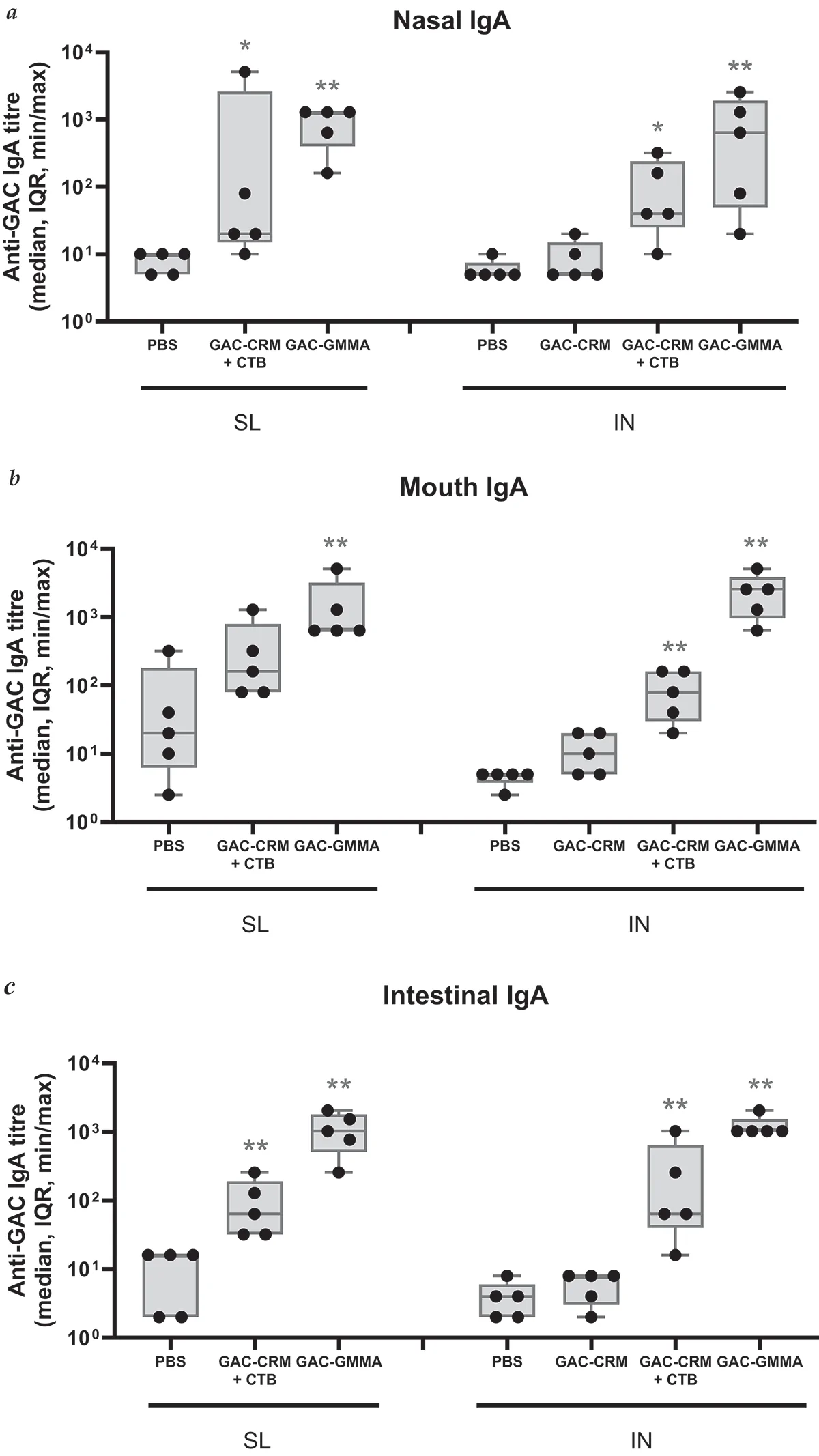
Comparison of mucosal IgA response following SL or IN immunization. Groups of mice (n = 5) were immunized sublingually or intranasally five times, at weekly intervals, with GAC-GMMA conjugate, GAC-CRM_197_ (with or without CTB), or with PBS. Individual animal responses are shown as dots. Horizontal lines indicate the median and boxes represent the interquartile range (IQR). Data is presented for IgA in nasal (a), mouth (b), intestinal (c), and vaginal (d) washes. Overall differences between groups were assessed using the Kruskal-Wallis test (*p* = .0003 for nasal IgA; and *p* < .0001 for mouth, intestinal and vaginal IgA). Significant differences with the PBS control group are indicated by *p < .05.

**Figure 5.**
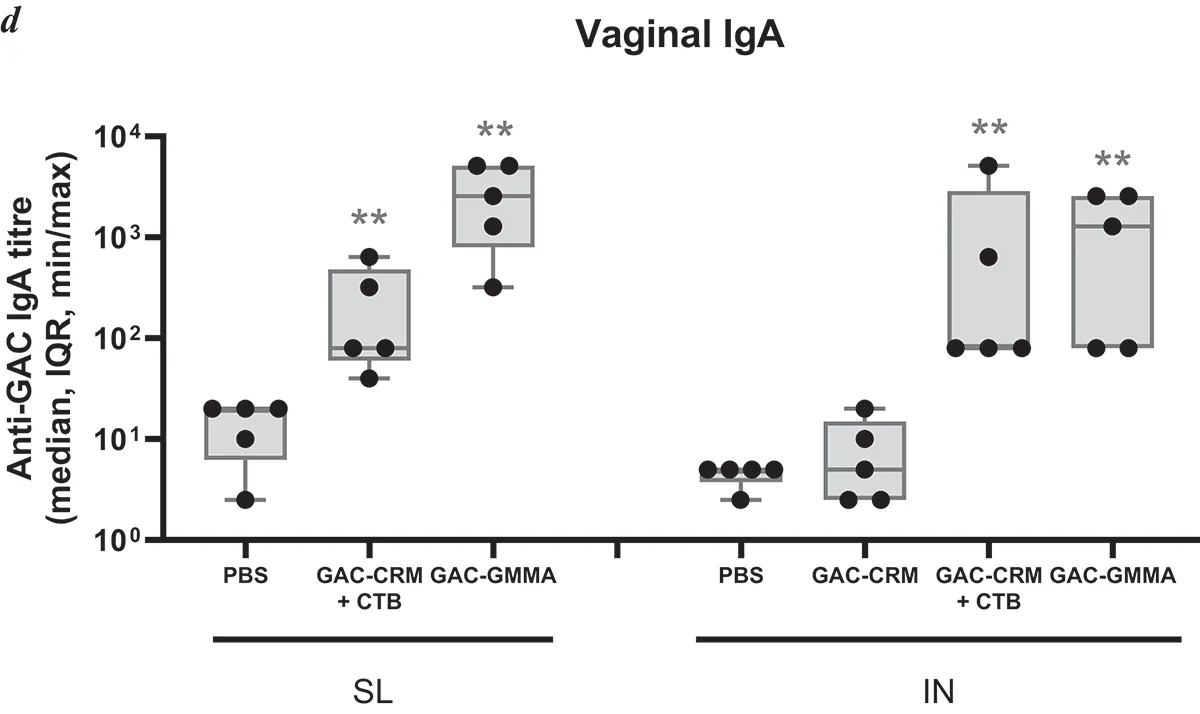
(Continued).

Intranasal administration of GAC-CRM_197_ with CTB similarly induced mucosal IgA responses comparable to those elicited by the SL route. Nasal IgA titers were 40 (40–160) and 20 (20–80) following IN and SL immunization, respectively, with no significance difference between routes ( = 0.754; rrb = −0.20). Oral IgA responses were also detected following both routes, with median titers of 80 (40–160) following IN immunization and 160 (80–320) following SL immunization. Although titers were numerically higher following SL administration, the difference was not statistically significant (*p* = .246; rrb = 0.20). Intestinal IgA responses were also comparable between routes, with median IgA titers of 64 (64–256) and 64 (32–128) following IN and SL immunization, respectively (*p* = .754; rrb = 0.12). Vaginal IgA titers were also similar following IN and SL administration, with median titers of 80 (80–640) and 80 (80–320), respectively, and no significant difference between routes (*p* = .643; rrb = 0.16).

Collectively, these findings demonstrate that GAC-GMMA is highly effective at inducing both local and distal mucosal immunity following intranasal administration.

### GMMA mediates induction of Th1 and Th17 responses in parenteral and mucosal immunization

To understand the nature of the T cell response induced by the GMMA and -CRM_197_ conjugates, we measured cytokine secretion by immune spleen cells from the SC, SL and IN immunized mice following a co-culture with the homologues conjugate. Results presented in Table 2 showed differences in the cytokines profiles between CRM_197_ and GMMA conjugates. In all routes of immunization for GMMA conjugates, spleen cell supernatants contained a mixture of IFNγ and IL-17, and no IL-4. Meanwhile, immune cells from CRM_197_ conjugate groups secreted a mixture of the IFNγ and IL-4, but no IL-17 following SC immunization in the presence of alum, and a mixture of the three cytokines (IFNγ, IL-4 and IL-17) following SL and IN immunization in the presence of CTB. IL-4 was the highest in the SC group immunized in the presence of alum. This indicates that GMMA mediated the induction of Th1 and Th17 responses in all routes of immunizations, while CRM_197_ conjugate induced a mixture of Th1 and Th2 following SC immunization and a mixture of Th1, Th2 and Th17 following SL and IN immunization in the presence of CTB.

**Table 2.** Cytokine concentrations in supernatant of immune spleen cells.

Cytokine concentration (pg/ml)									
	IFNγ			IL-4			IL-17		
Conjugate	SC	SL	IN	SC	SL	IN	SC	SL	IN
GAC-CRM_197_ +Alhydrogel/CTB	156	258	226	53	7	6	9	665	410
GAC-GMMA	268	83	181	0	0	0	415	260	565

Spleen cells from immunized mice were cultured in 24-well plate for 2 d in the presence of 5 µg/ml of GAC-CRM197 or GAC-GMMA in a total volume of 2 ml of culture medium (as described in M&M). At 48 hours, the culture supernatant was harvested and levels of IL-4, IFNγ and IL-17 were measured by standard sandwich ELISAs. The concentration of each cytokine was calculated relative to a mouse cytokine reference standard tested on the same plate. Samples were tested in duplicates and results presented as mean of duplicates.

### Serum IgG and mucosal IgA bind to GAS strains

Immunofluorescence staining of GAS M1 strain with immune sera and mucosal secretions for IgG and IgA antibodies, respectively, was used to show IgG and IgA binding to the surface of GAS. Intestinal washes were used as a surrogate for mucosal secretions due to the larger sample volume available for analysis.

Data presented in Figure 6 indicated that IgG in immune sera and IgA in intestinal washes from GMMA or CRM_197_ conjugate groups bound to M1 strain, except for intestinal washes from SL delivery of CRM_197_ conjugate which showed no IgA binding above that of the PBS group. In general, and corroborating the ELISA data (Figures 4 and 5), GMMA conjugates elicited serum IgG and mucosal IgA antibodies that consistently showed superior binding to the M1 strain than those of the CRM_197_ conjugate groups. As expected, no IgA binding was seen from SC immunization which had not generated any mucosal IgA (Figure 6).

**Figure 6.**
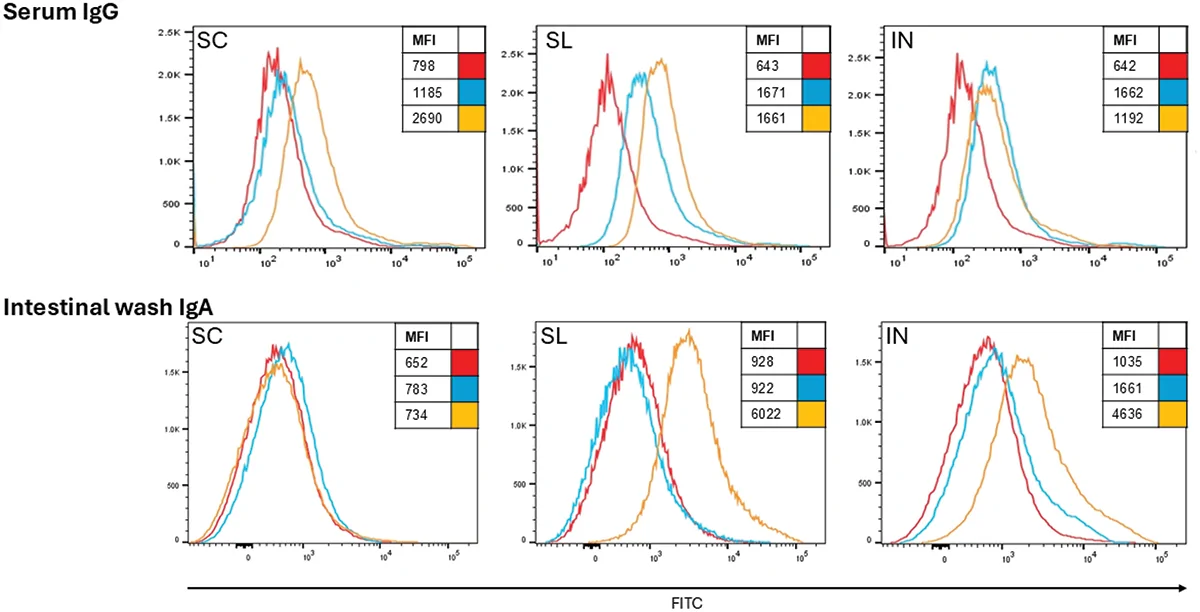
Immunofluorescence staining of GAS M1 strain with immune mouse serum or intestinal washes. GAS UK M1 strain was stained with immune sera or intestinal washes (as a surrogate for mucosal secretions) from mice immunized SC, SL or IN with GAC-GMMA (yellow), GAC-CRM_197_+CTB (blue), or with PBS (red) as a negative control. Antibody binding was shown as a shift in fluorescence intensity signal above the PBS group.

As Figure 6 shows, there was a clear shift in FITC signal in M1 GAS stained with immune serum IgG and mucosal (intestinal) IgA from GAC-CRM_197_ -adjuvanted and GAC-GMMA immunized groups compared to the PBS control group. It was also evident that the fluorescence intensity of the staining was much higher for the GMMA groups compared to the CRM_197_ groups for both systemic IgG and mucosal IgA, except for serum IgG following IN, where the CRM_197_ group had a slightly higher signal than the GMMA group, probably due to the higher IgG level induced by the CRM_197_ group.

## Discussion

This study evaluated the immunogenicity of a GMMA-based glycoconjugate vaccine targeting GAS following mucosal delivery via SL and IN routes in a mouse model. The results demonstrate that GMMA can function as both a carrier and an intrinsic adjuvant, inducing robust systemic IgG responses alongside mucosal IgA responses at multiple mucosal sites, without the need for an additional adjuvant.

Parenterally administered conjugate vaccines have been highly successful in reducing both disease and mucosal colonization for several bacterial pathogens, primarily through the induction of IgG responses.^29–31^ This present study does not aim to demonstrate the superiority of mucosal vaccination over parenteral immunization but instead provides a preclinical evaluation of the ability of GMMA-based conjugate formulations to induce combined systemic and mucosal immune responses when administered via mucosal routes. Mucosal surfaces are the main point of entry for most human pathogens; hence, induction of immunity at those surfaces is of paramount importance to prevent infection and to further reduce transmission of pathogens from one person to another.^35^ In a previous study of a GAC conjugate, intranasal vaccination of mice demonstrated reduced oral colonization,^14^ suggesting a use of mucosal vaccination for this particular pathogen. Compared with traditional injection-based vaccination, mucosal delivery methods, like natural infection, can facilitate the interaction of antigens with mucosa-associated lymphoid tissue (MALT) to effectively induce both mucosal and systemic immunity.^17^ Several strategies have been used for mucosal vaccine delivery, including oral delivery (using pills, liquid, or small patches), intranasal and respiratory delivery (using nebulizers, nasal spray, and inhalers).^36^

Although numerous effective mucosal vaccines are in use, they are mainly live attenuated and inactivated whole-cell preparations, while most subunit vaccines are, in general, weakly immunogenic when administered by the mucosal routes and require the use of a potent mucosal adjuvant and/or delivery system to generate a robust immune response.^35^ This suggests that advancing mucosal vaccine delivery requires innovative adjuvant approaches and delivery strategies.

Results from our previous studies using the SC delivery route demonstrated the ability of GMMA to work as a good carrier, as well as an adjuvant, for GAC and mediated the induction of a substantial anti-GAC IgG response similar to or better than that induced by alum-adjuvanted GAC-CRM_197_ conjugate, both in rabbits and mice.^28^ Therefore, we were interested in investigating if GMMA as a carrier also has mucosal self-adjuvanticity properties to promote induction of systemic and mucosal immunity against GAS following delivery of GAC-GMMA conjugate by the SL and IN routes. Our results showed that mucosal delivery of GAC-GMMA (without any additional adjuvant) mediated the induction of anti-GAC serum IgG and mucosal IgA antibodies at the immunization sites (mouth and nose) and at other distant mucosal sites, e.g. intestines and vagina. This indicated that mucosal immunization with GMMA conjugate does not require any additional adjuvant for mucosal delivery. In contrast, although the classical GAC-CRM_197_ conjugate was also able to induce substantial systemic and mucosal responses at local and distant mucosal sites, this was only when it was co-administered with a potent adjuvant (CTB). In addition, induction of mucosal IgA at local and distant mucosal surfaces is in line with other studies which demonstrated that, although mucosal immune responses are, in general, compartmentalized, there is a crosstalk between the different mucosae, and it is thus possible to vaccinate at a single mucosal site but also promote immune responses at distant mucosal surfaces.^37^

In addition, our results showed that the addition of the known mucosal adjuvant CTB to GMMA conjugate did not show any synergistic effect but instead had a slight, though not significant, negative impact on the IgG and IgA anti-GAC responses, possibly as a result of interfering mechanism(s). Similar results were recently reported by Mancini et al.^38^ where the addition of neither Alhydrogel nor AS37 improved the magnitude or the functionality of *S. sonnei* GMMA vaccine in mice and rabbits.

Mucosal adjuvants can be divided into two categories: 1- immune stimulants e.g. heat-labile enterotoxin (LT), CT, TLR ligands, e.g. CPG oligodeoxynucleotides, poly(I:C) and monophosphoryl lipid A, cytokines and chemokines that can activate innate immunity, and 2- immune delivery systems (e.g. virus-like particles, liposomes, polymer-based nanoparticles, lipid-based nanocarriers and others) which can protect the antigens from harsh conditions and target it to M cells, dendritic cells and other antigen presenting cells at mucosal inductive sites.^39,40^ Our results showed that while both GMMA and CTB seem to be powerful adjuvants for the generation of mucosal and systemic immunity, their mode of actions seems to be different as well due to the type of Th cell response they induce. This was clear in our results where the subtype of the systemic IgG response induced by the two conjugates was different, with GMMA conjugate mediating the induction of a mixture of Th1 and Th2 response (IgG1 and IgG2a with a bias toward IgG2a), white CTB-adjuvanted CRM_197_ conjugate induced mainly a Th2 response (IgG1 with very little IgG2a). These findings were similar to what we have previously reported from SC immunization with GAC-GMMA and GAC-CRM_197_+ alum.^28^ It is well known that adjuvants induce changes in the Th1-Th2 balance, with CTB (similar to Aluminum adjuvants) inducing a Th2 bias response to HIV-DNA intramuscular immunization in mice.^41^ While mucosal IgA is often considered a key mediator of protection at mucosal surfaces, evidence from licensed vaccines is controversial. While some studies indicates that serum IgG plays a central role in protection against many bacterial pathogens, including those colonizing mucosal tissues,^42^ other studies demonstrated a role for secretory IgA in reducing the risk of viral transmission and viral shedding in the respiratory tract, indicating a strong correlation between early IgA response and viral clearance.^43^ Currently no correlate of protection is known for GAS, despite extensive studies on human IgG responses, therefore there may be synergy between secretory IgA and systemic IgG responses.^33^ The findings presented here should therefore be interpreted within this broader context, where mucosal immune responses may complement, rather than replace, systemic immunity. Therefore, the induction of mucosal IgA observed in this study may contribute to pathogen neutralization at mucosal surfaces by preventing microbial attachment to epithelial cells. However, its relative contribution to protection against GAS infection in humans remains to be determined. The reported results of high level of IgG2a combined with mucosal IgA following immunization with the GAC-GMMA conjugate possibly suggests a high level of systemic and mucosal protection against GAS infection, as it is well known that the four mouse IgG isotypes differ extensively in bactericidal and opsonophagocytic activity with IgG2a being one of the most effective mediators of opsonophagocytic activity, owing to its high-affinity interactions with activating Fcγ receptors on phagocytes and activation of phagocytes and NK cells.^44,45^ This is in addition to the potential of immune exclusion at mucosal surfaces mediated by the anti-GAC IgA response.

The adjuvanticity of CTB is mediated by its high affinity binding to monoganglioside GM1 receptor. The fact that CTB is non-toxic has made this molecule an excellent delivery vehicle, given the cellular distribution of its receptor. GM1 is found on a broad variety of cell types, including epithelial cells and on antigen presenting cells such as macrophages, dendritic cells and B cells. Binding of CTB to its receptor increases expression of MHC class II molecules and activation molecules on APCs, which results in the secretion of cytokines and chemokines, thereby enhancing immune responses.^46,47^ GMMA resemble the outer membrane vesicles (OMV) of the bacterial pathogen they shed and have been shown to be an effective carrier and adjuvant, enhancing immunogenicity of proteins and carbohydrates.^28,48^ It displays several pathogen associated molecular patterns (PAMPs), which are recognized by pattern recognition receptors (PRRs) expressed on mammalian cells,^49^ including lipopolysaccharides and lipoproteins that are TLR-2 and TLR-4 activators.^50,51^ PAMPs’ interaction with PRRs rapidly activates the innate immune system, with the induction of pro-inflammatory cytokines and chemokines, and that may be the basis of GMMA self-adjuvanticity, in addition to its nano-sized particle properties (50–200 nm) which are capable of direct diffusion into draining lymph nodes and improve antigen uptake by APCs. Our results demonstrate for the first time that GMMA adjuvanticity is also extended to facilitate induction of mucosal antibody responses at multiple mucosal sites without the need for an additional adjuvant, as is the case for classical conjugate vaccines.

In this study we showed that mucosal (SL and IN), but not parenteral, delivery of GAC conjugate vaccines induced expression of IL-17 which is known to be induced by mucosal infection or vaccines administered by mucosal rather than parenteral routes. In addition to its protective role, IL-17 is well known to synergize with IFNγ, which is also produced by all routes used in this study, to enhance nitric oxide production by macrophages and, thereby promoting protection at mucosal surfaces.^52^

Opsonophagocytosis killing assays (OPKAs) for GAS against non-M-protein antigens are not currently standardized, and although a THP-1 assay has been developed and demonstrated with GAC‑coated fluorescent beads, this does not show killing activity.^53^ However, binding of immune antibodies to bacteria is the first step in initiating bacterial killing (mediated by IgG) or immune exclusion at mucosal surfaces (mediated by secretory IgA).^54,55^ Our results from immunofluorescence staining showed that both IgG and IgA anti-GAC antibodies were able to bind to a GAS M1 strain, with those elicited by the GMMA conjugate giving much higher signals than CRM_197_ adjuvanted conjugate (except for IgG following IN immunization, where the intensity of the staining was similar), irrespective of the route of immunization, suggesting the superiority of the GMMA as an adjuvant. Interestingly, even when the serum IgG response was slightly higher for the CRM_197_ conjugate group after IN immunization, the FITC signal was similar for the GMMA and the CRM_197_ conjugate groups, suggesting higher affinity of the GMMA-induced antibodies. However, further studies to investigate the ability of the immune response to either kill GAS or prevent colonization are needed.

While some studies reported the superiority of the intranasal over the sublingual route, possibly as a consequence of the differences in mucosal tissues between the two routes,^56^ our results showed that both SL and IN delivery of GAC-GMMA were able to induce similar systemic and mucosal immunity at local and distal mucosal sites. However, each of the two routes has advantages and disadvantages. While intranasal delivery takes advantage of the presence of the nasal associated lymphoid tissue, it may pose a risk redirecting antigen or adjuvant to the central nervous system.^57,58^ Although the sublingual mucosa is devoid of M cells, induction of local and disseminated immune response after SL immunization relies on resident DCs to sample antigens and to migrate to local draining lymph nodes for initiation of a local response and to distant secondary lymphoid organs, in addition to selective migration of IgA antibody secreting cells to distant mucosal sites using chemokine/chemokine receptor system.^59^

Our results from the mouse model provide a controlled environment to investigate immune responses following mucosal routes of administration of GAC-GMMA conjugates, even where direct translation to human vaccination strategies is uncertain. However, a limitation of this study is the use of anesthesia during sublingual and intranasal immunization, which does not directly reflect human vaccination conditions. Anesthesia is commonly required in small animal models to ensure accurate delivery and retention of vaccine formulations at mucosal sites. However, this may influence antigen exposure and immune responses and therefore limit the direct translational relevance of the findings to the human situation.

The main constraint of the study is the lack of data from a pathogen challenge model to assess protection against GAS colonization or infection following mucosal immunization and therefore conclusions are limited to immunogenicity. In addition, the duration of the immune response, particularly the persistence of mucosal IgA, was not evaluated. Furthermore, the study explored a limited number of vaccine doses and did not include comprehensive optimization of dose volume or schedule. Future studies should include evaluation in appropriate challenge models to assess the impact of GMMA-based vaccination on GAS colonization and infection. Investigations into the durability of the immune response, as well as mechanistic studies examining the pathways involved in GMMA-mediated immune activation, will further enhance understanding of this platform.

## Conclusions

In conclusion, this study demonstrates that GMMA-based glycoconjugate vaccines can induce both systemic and mucosal immune responses following mucosal administration in a mouse model. Taken together, our results demonstrate that GMMA contribute advantages that extend beyond their role as a carrier for GAC. When delivered by either the SL or IN route, GMMA-based conjugates were able to elicit strong mucosal and systemic anti-GAC antibody responses, along with cellular immunity characterized by Th1 and Th17 signatures. These properties underline the suitability of GMMA-based formulations for vaccines that aim to prevent infections at mucosal surfaces, such as GAS. While the translational relevance of mucosal vaccination strategies for bacterial pathogens in humans remains to be fully established, these findings contribute to the understanding of GMMA as a flexible vaccine platform and support further investigation into its potential applications. In addition, the practical advantages of mucosal delivery, which requires simple, low-cost methods that can be readily deployed using simple and low-cost delivery methods, make this approach particularly promising for vaccination programs in resource-limited regions, including low- and middle-income countries.

## Data Availability

The raw data supporting the conclusions of this article will be made available by the corresponding author on request.
